# Supplementary material for: Peptide Vaccination against Cytomegalovirus Induces Specific T Cell Response in Responses in CMV Seronegative End-Stage Renal Disease Patients
Source: Vaccines (Basel). 2021 Feb 6;9(2):133. doi: 10.3390/vaccines9020133 (PMC7915922; doi:10.3390/vaccines9020133)
Supplement: Supplementary file 1 [file vaccines-09-00133-s001.pdf]

Supplementary Table S1. Antibody list.

Name	Dye	Clone	Cat.NO.	Company
CD3	V450	UCHT1	560366	BD
CD8	FITC	SK1	344704	Biolegend
CD14	PerCP	HCD14	325632	Biolegend
CD19	PerCP	HIB19	302228	Biolegend
CD45RA	APC	HI100	304112	Biolegend
CCR7	PE-Cy7	3D12	25-1979-42	eBioscience

Abbreviations: Cat. NO. = catalogue number; PerCP = peridinin chlorophyll; FITC = fluorescein isothiocyanate; APC = allophycocyanin; PE = phycoerythrin; PE-Cy7 = phycoerythrin-Cy7.

Spplementary Table S2. Release Criteria for peptide vaccines.

	**Specification**	**Mean value (± SD)**
Volume	1.400 µl ≙ 1.400 g ± 5 %	1.414 g (± 0.033 g)
Peptide content	300 µg ± 20%<br>(C.A.T. GmbH & Co, Tübingen)	324.1 µg (± 25.76 µg)
Sterility	Sterility testing according to Ph. Eur. 2.6.1.<br>(L+S AG, Bad Bocklet)	sterile (all products)

Abbreviations: Ph. Eur. = Pharmacopoeia European
